# Fatigue Performance of Different Thickness Structure Combinations of Hot Mix Asphalt and Cement Emulsified Asphalt Mixtures

**DOI:** 10.3390/ma11071145

**Published:** 2018-07-05

**Authors:** Zhenjun Wang, Linlin Cai, Xiaofeng Wang, Chuang Xu, Bo Yang, Jingjing Xiao

**Affiliations:** 1 School of Materials Science and Engineering, Chang’an University, Xi’an 710061, China; xc_chd029cico@163.com; 2 Henan Provincial Communications Planning & Design Institute Co., Ltd., Zhengzhou 450052, China; wangxf0351@sina.com (X.W.); yangbohnrbi@foxmail.com (B.Y.); 3 Engineering Research Central of Pavement Materials, Ministry of Education of China, Chang’an University, Xi’an 710061, China; xiaojj029@sina.com

**Keywords:** cement emulsified asphalt mixture, fatigue performance, thickness combinations, X-ray computed tomography, artificial neural network

## Abstract

Cement emulsified asphalt mixture (CEAM) is widely used in asphalt pavement for its environmental virtues. However, a CEAM layer can influence fatigue performance of asphalt pavement because of higher air voids of CEAM in contrast to hot mix asphalt (HMA). Therefore, it is common to use HMA and CEAM structure combinations for improving the fatigue performance. In this work, three different thickness structure combinations of HMA (AC-10) and CEAM (AC-16) were designed, in which HMA and CEAM were used as top layer and bottom layer, respectively. The fatigue performance of the three combinations was studied. The fatigue equations of the combinations were established and the rational combination was recommended. The distributions of the internal voids in the combinations were studied with X-ray computed tomography (X-ray CT); and the correlation between the fatigue life and the void ratios were analyzed. Artificial neural network (ANN) was employed to predict the fatigue life of each combination. The results show that the fatigue life of the combinations is inversely proportional to the stress ratio level and environment temperature. The optimal combination is the structure with 40 mm HMA and 40 mm CEAM. The internal void ratio of CEAM is higher than that of HMA. A thinner HMA and thicker CEAM structure can result in higher void ratios and lower fatigue life of the combinations. The prediction results of ANN are similar to the experimental results. The obtained results can potentially guide the design of cement emulsified asphalt pavement structures.

## 1. Introduction

### 1.1. Background

Cement emulsified asphalt mixture (CEAM) consists of asphalt emulsion, cement, aggregate, and mineral fillers, which is widely used in road construction and maintenance for environmental requirements. CEAM has many advantages such as low pollution, low energy consumption, low cost, and easy construction [1,2]. However, the loss of cohesive force in its internal structure causes the CEAM mixture to be poor in water resistance, forming a pit, crack, and other pavement damage. Therefore, CEAM cannot be applied to the road surface alone [3]. On the contrary, hot mix asphalt (HMA) has been widely used in pavement structure because of its high and low temperature performance and high cohesive force [4]. However, the problems such as high pollution, high energy consumption, and high cost caused by HMA cannot be ignored. Therefore, the advantage combination of cement emulsified asphalt mixture and ordinary hot mix asphalt mixture in asphalt pavement is important for asphalt pavement design.

With the rapid development of transportation, the asphalt pavement is facing more and more severe fatigue damages. Previous studies have concentrated on the fatigue performance materials varied with stress ratios, asphalt contents, void ratios, aggregate types, and so on. For example, Li, et al. [5,6] found that the fatigue life of the mixture increased with the increase of asphalt content at the high stress ratio level, and the cement content can improve the fatigue life at the low stress ratio level. Wang, Cory, and Chiara, et al. [7,8,9] reported that the factors for the fatigue performance of CEAM can be sorted in their influence as emulsified asphalt content > stress ratio level > cement content. Jiang, et al. [10] pointed out that the fatigue life of the asphalt mixture decreases with the increase of void ratios. Chen, Wu, and Gao, et al. [11,12,13] indicated that the rougher aggregate surface was beneficial to increase the fatigue life of the asphalt mixture; and the fatigue damage tended to occur in areas where more aggregates were concentrated.

The asphalt pavement structure design is facing more and more fatigue failure challenges [4,14]. To predict the fatigue life of the asphalt mixture, scholars have studied the correlation between the vehicle repeated loading times and the mechanical response of the asphalt pavement [15]. Three main research methods for the fatigue characteristics of the asphalt pavement were developed as follows: (1) phenomenological method—a method for characterizing the fatigue fracture characteristics of materials using fatigue life curves [16]; (2) mechanics approximation method—the stress intensity factor, which was generated at the crack tip of the pavement, was used to study the development law of various cracks when the material undergoes fatigue failure under repeated loading [17]; and (3) energy dissipation method—a method for characterizing the fatigue life of the asphalt mixture with the energy accumulation value when the mixture reached failure under a certain cyclic load. The dissipated energy can not only analyze the damage process of the internal structure in a theoretical basis, but also qualitatively study the fatigue performance of the asphalt mixture [18,19]. However, the existing research mainly focuses on the fatigue performances of the HMA mixture as a single surface layer.

### 1.2. Objectives

CEAM is often used in the middle or upper layers of asphalt pavement structure. There are evident differences in the properties of CEAM and HMA, such as air void and flexural tensile strength. However, less attention is paid to the fatigue performance of different asphalt pavement thickness structure combinations. That is to say, it is rare to find studies concentrating on the fatigue performance of the asphalt pavement layer with various thickness structure combinations of HMA and CEAM. Therefore, three kinds of thickness structure combinations with HMA and CEAM were prepared in this work, in which the top layer was HMA and the bottom layer was CEAM. The effects of thickness combinations on the fatigue performance of the structures were evaluated under different temperatures and stress levels. Analyses between the internal void distributions and fatigue life were carried out with an X-ray computed tomography (X-ray CT) technique. Based on the experimental results, an artificial neural network (ANN) model was developed to predict the fatigue life of the different thickness structure combinations. The results of this work can contribute to putting CEAM to use reasonably in asphalt pavement structures. The conclusions can also be potentially used to guide the asphalt pavement structure design with CEAM and HMA mixtures.

## 2. Experimental

### 2.1. Raw Materials

The binder of HMA was asphalt and its properties are shown in Table 1. Ordinary Portland cement was used in CEAM and its main properties are given in Table 2. Cationic emulsified asphalt was adopted in CEAM and its properties are shown in Table 3. Limestone aggregate and mineral fillers were used in two kinds of the mixtures, and their properties are shown in Table 4 and Table 5.

### 2.2. Preparation of the Specimens

**Figure 1:**
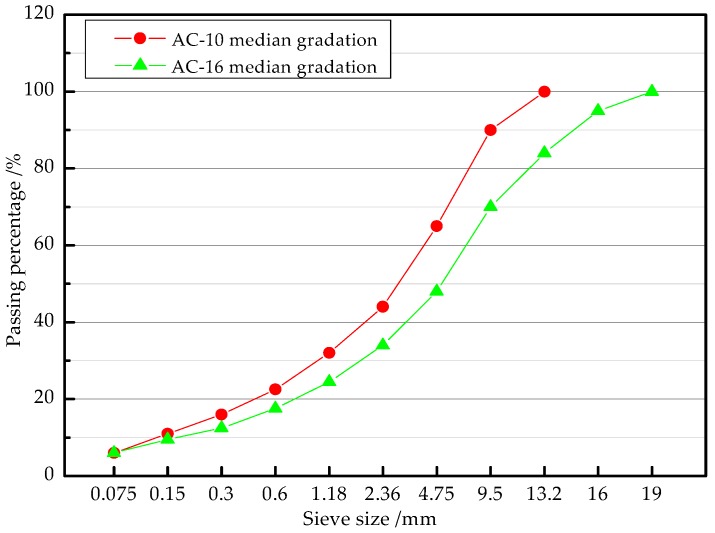
Aggregate gradations of hot mix asphalt (HMA) and cement emulsified asphalt mixture (CEAM).

In accordance with the specification [22], the aggregate gradations for three different thickness structure combinations of HMA (AC-10) and CEAM (AC-16) are shown in Figure 1. The HMA and CEAM mixtures were prepared in reference to the specifications [23]. The properties of the mixtures were tested and the results are shown in Table 6 and Table 7. Each specimen was prepared with two layers, which were the top layer and bottom layer. The total structure thickness of each specimen remained at 80 mm. AC-10 HMA was used in the top layer. Its thickness was designed as 40 mm, 30 mm, and 20 mm. Meanwhile, CEAM was used in the bottom layer. Its thickness was designed as 40 mm, 50 mm, and 60 mm.

**Figure 2:**
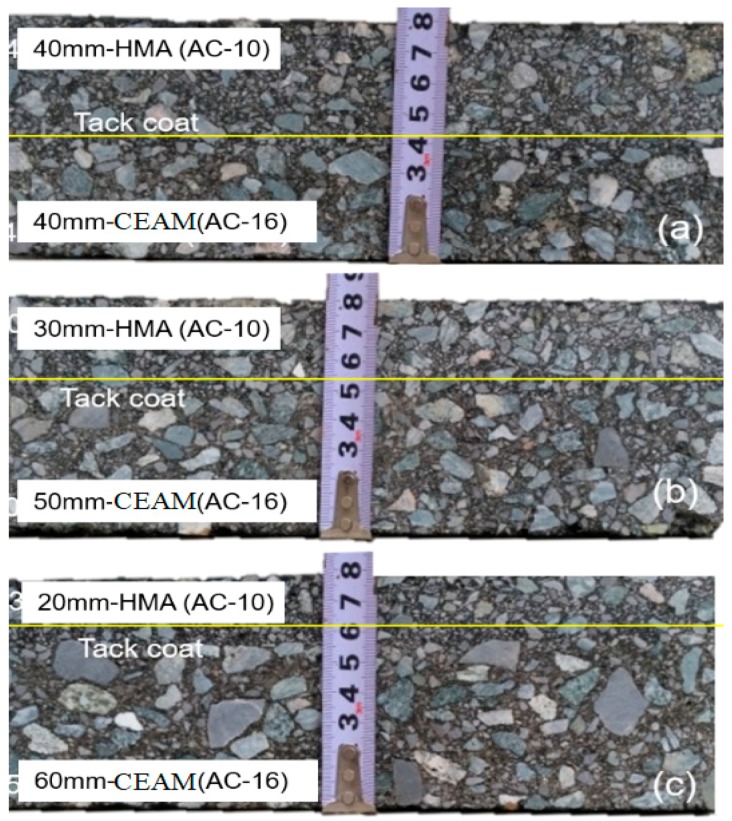
Pictures of different thickness structure combinations: (**a**) C1; (**b**) C2; and (**c**) C3.

The bottom layer, CEAM, was compacted by the wheel forming machine (LCX, Beijing, China) with 15 round-trips (30 times) at the temperature of 25 °C. Then, the emulsified asphalt was sprayed as the tack coat layer. The HAM mixture was paved as the upper layer after demulsification of the emulsified asphalt and the HAM mixture was compacted by the wheel machine (LCX, Beijing, China) with 15 round-trips (30 times) at the temperature of 150 °C. Therefore, three thickness structure combinations, with sizes of 300 mm × 200 mm × 80 mm, were prepared as 40 mm (HMA) + 40 mm (CEAM) (marked as C1); 30 mm (HMA) + 50 mm (CEAM) (marked as C2); and 20 mm (HMA) + 60 mm (CEAM) (marked as C3). Finally, the thickness structure combination specimens were cured at the temperature of 25 °C for 28 days, so that the CEAM can possess enough mechanical performance. Afterwards, the specimens were sawed to the trabecular specimens with sizes of 250 mm × 50 mm × 80 mm for the fatigue tests, which are shown in Figure 2.

### 2.3. Test Methods

#### 2.3.1. Flexural Tensile Strength Test

**Figure 3:**
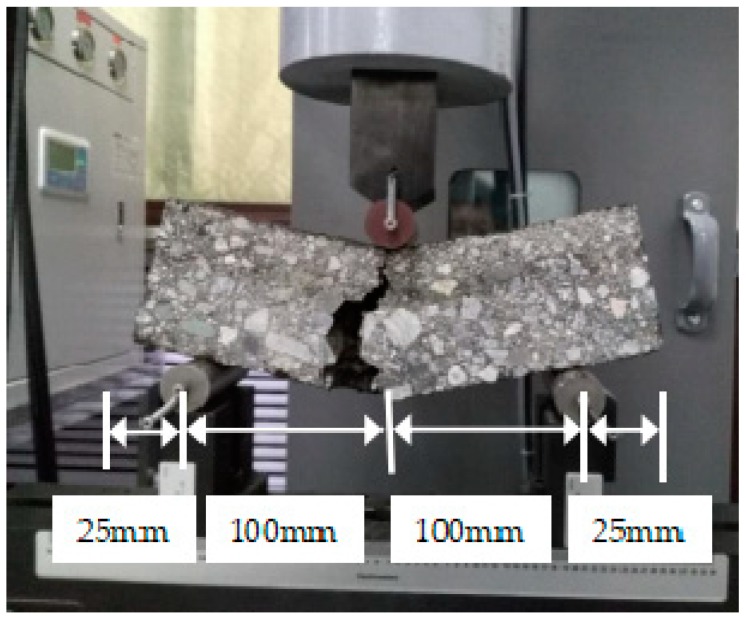
The setup for flexural tensile strength test.

The material testing system (MTS-370, MTS Systems Corporation, MN, USA) was used for the flexural tensile strength test at the temperature of 20 °C. The preload was conducted with a cyclic load of approximately 1.0 kN to prevent the poor touch between the indenter and the test piece. The loading speed for the bending strength test was 50 mm/min, which was slowly loaded on the center of the specimen to break it. All of the tests were carried out three times and the average values were used as the testing result. The maximum deflection of the specimen was recorded. The setup for the flexural tensile strength test is shown in Figure 3 and the flexural tensile strength and the flexural stiffness module of the three combinations were calculated by Equations (1) to (3).

(1) $$R_{B}=\frac{3LP_{B}}{2bh^{2}}$$

(2) $$\varepsilon_{B}=\frac{6hd}{L^{2}}$$

(3) $$S_{B}=\frac{R_{B}}{\varepsilon_{B}}$$

where, $R_{B}$ is flexural tensile strength, MPa; L is the distances between two support points, mm; $P_{B}$ is the load, N; b and h are the width and height of the specimen, mm, respectively; d is the maximum deflection, mm; $\varepsilon_{B}$ is the bending strain, no unit; and $S_{B}$ is the flexural stiffness modulus, no unit.

#### 2.3.2. Fatigue Test

The fatigue life *N_f_* of different thickness structure combinations was tested by the three-point bending load fatigue test under the controlled stress loading mode, which is more suitable for the thicker asphalt mixture specimens, with a thickness of 80 mm in this work, and is easy to control in fatigue test. The loading waveform was a continuous full sinusoid wave with a fixed frequency of 10.0 Hz. The material testing system (MTS-370, MTS Systems Corporation, MN, USA) was used to perform the bending fatigue test. The test stress levels were 0.3, 0.4, and 0.5, respectively. The test temperature was 5 °C, 15 °C, and 25 °C, respectively. The axial displacement during the loading process at the temperature of 15 °C was recorded with the increase of the loading time. All of the tests were carried out three times.

#### 2.3.3. X-ray CT Scanning

The industrial X-ray CT machine (YXLON Compact-225, Hamburg, Germany) was adopted to obtain the image of the internal air voids in mixtures to analyze the distribution characteristics of the voids. The parameters of the industrial X-ray CT are shown in Table 8. The scanning space for the combination was 0.01 m. The trabecular specimen with a thickness combination of 40 mm + 40 mm was scanned by CT scanning from the left to the right, with a total of 900 images; and the 180th, 360th, 540th, and 720th images were selected for processing and analyses, which lay at the position of 50 mm, 100 mm, 150 mm, and 200 mm of the specimen. The higher the density, the greater the brightness of the image. The image tended to be white. The lower the density, the smaller the brightness of the image. The image tended to be black. The void ratios were obtained by measuring the grayscale distribution of the images.

#### 2.3.4. Fatigue Life Prediction of the Combinations Based on ANN

This work developed an ANN for predicting the fatigue lives of different combinations under various conditions, which were not tested by the three-point bending load fatigue test. The main processes for developing an ANN included three steps, namely: (a) pre-treat results of the fatigue tests; (b) training an ANN model using a feed-forward algorithm; and (c) testing the precision of the ANN.

**Figure 4:**
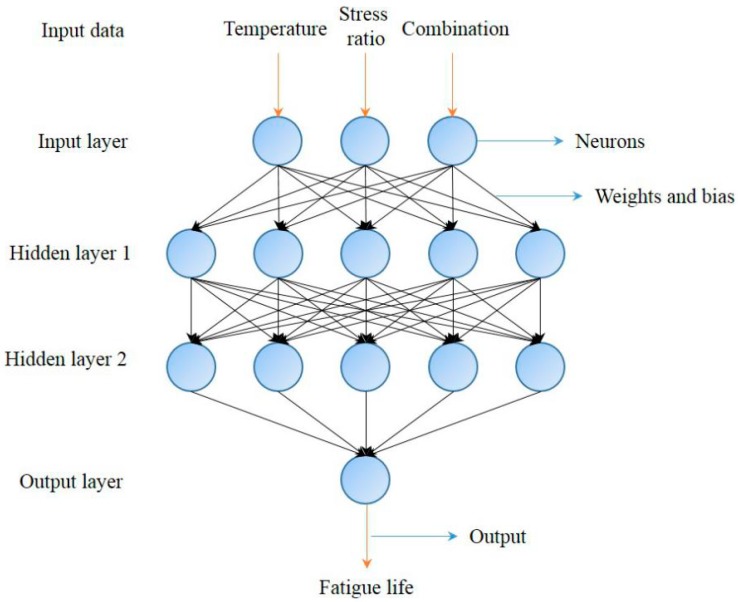
Network structure model of the mixtures.

The network structure of the ANN for predicting the fatigue life is shown in Figure 4. As shown in Figure 4, the ANN consists of an input layer, two hidden layers, and an output layer. The size of the input layer is 3 × 1, which means three factors of fatigue life are inputted to the ANN, including the temperature, stress level, and thickness combination. Both of the two hidden layers consist of five neurons. The double-layer structure of the hidden layers and the number of neurons can predict the output precision of the ANN. The size of output layer is 1 × 1, which means that the output of the ANN is the fatigue life.

The first step for developing the ANN to predict the fatigue life is to pretreat the input data and target data. The input data of the ANN contain the temperature, stress level, and thickness combinations. Different thickness combinations of the ANN and the thickness of the top layer are utilized to represent the characters. For example, as a C2 structure combination was 30 mm + 50 mm, the input data of the thickness characteristic of C2 is 50. Then, the temperature data are normalized to eliminate the effect of the dimensions of these three factors and to avoid over fitting. The temperature value is divided by 40. Thus, 5 °C, 15 °C, and 25 °C can be normalized to 0.125, 0.375, and 0.625, respectively. Therefore, the input data of C2 in a 0.3 stress ratio level and 5 °C are (0.125, 0.3, 50). The target data of the ANN are the fatigue life of the combination in different conditions. Then, data of the fatigue life are normalized to eliminate the effect of the dimensions and to avoid over fitting. The fatigue life is divided by 40,000. For example, the fatigue life of C2 at 0.3 stress level and at 5 °C is 28,153 times. Thus, the normalization result is 0.704. Therefore, the target data of C2 at 0.3 stress level and 5 °C is 0.704.

The second step for developing the ANN to predict the fatigue life is to train and to test the ANN using a feed-forward algorithm. Detailed information on the feed-forward algorithm can be found in reference [24]. Training, testing, and target data for developing the ANN are a normalization results of the fatigue test. The last step is to predict fatigue life of different combination under various conditions by the well-trained ANN.

## 3. Results and Discussion

### 3.1. Results of Flexural Tensile Strength Test

**Figure 5:**
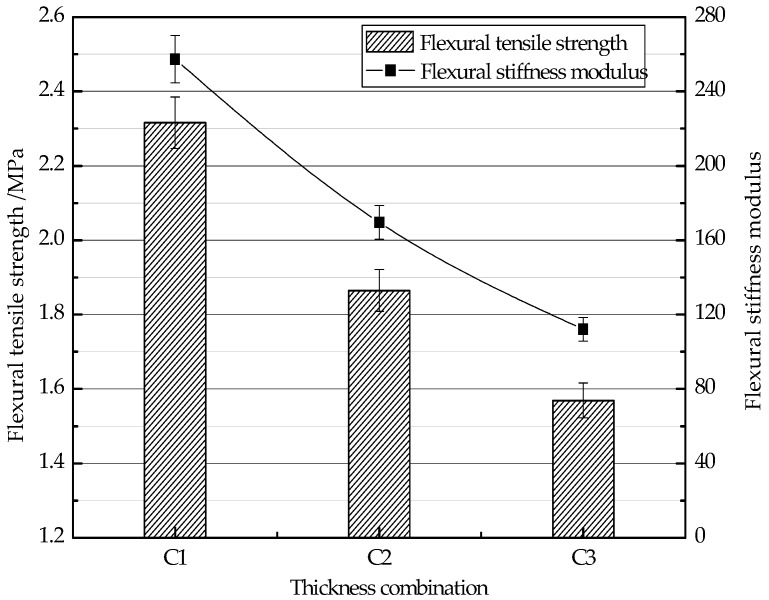
Flexural tensile strength test results of different thickness structure combinations.

The test results of flexural tensile strength *R_B_* and the flexural stiffness modulus *S_B_* of three thickness structure combinations are shown in Figure 5. When the thickness of CEAM increases from 40 mm to 60 mm and HMA decreases from 40 mm to 20 mm, the flexural tensile strength *R_B_* and the flexural stiffness modulus *S_B_* are dropped 32.2% and 56.4%, respectively. The values of the flexural tensile strength *R_B_* and the flexural stiffness modulus *S_B_* of the C1 combination are the highest. The bending strain *ε_B_* decreased with the thinner CEAM and the thicker HMA. That is because the AC-10 HMA as the material of the top layer is a viscoelastic material with high ductility and anti-deformation ability. Therefore, the thicker HMA layer and thinner CEAM layer in combination are propitious to enhance the mechanical performance of the thickness structure combinations under the bending force.

### 3.2. Effects of Test Temperature on Fatigue Performance

**Figure 6:**
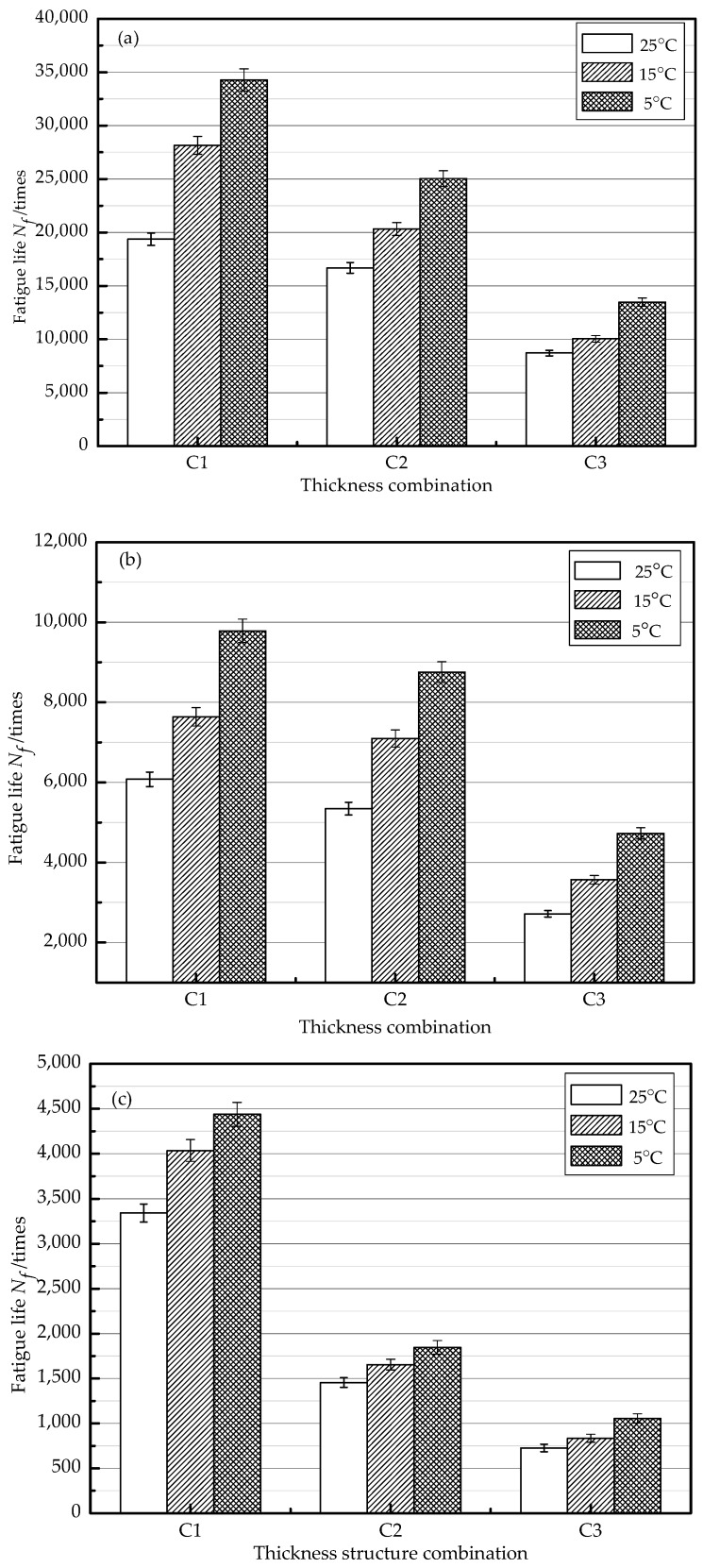
Fatigue life of different thickness structure combinations at different stress levels: (**a**) stress level 0.3; (**b**) stress level 0.4; and (**c**) stress level 0.5.

Figure 6 shows the effect of temperatures on the fatigue life of each thickness structure combination at different stress levels. The finding is that the fatigue life of all of the combinations increases with the decrease of the temperature under the same stress level. The reason lies in that the stiffness modulus of the asphalt mixtures increase when the temperature decreases within a certain range. In the meantime, strains of the combinations decrease continuously under the same cyclic load stress. The decrement of stiffness at the lower layer and the increment of thicknesses at the top layer can increase the total stiffness modulus. Therefore, the anti-cracking ability of the combinations has been enhanced; thus, the fatigue life of the combinations can be prolonged.

### 3.3. Effect of Stress Level on Fatigue Performance

**Figure 7:**
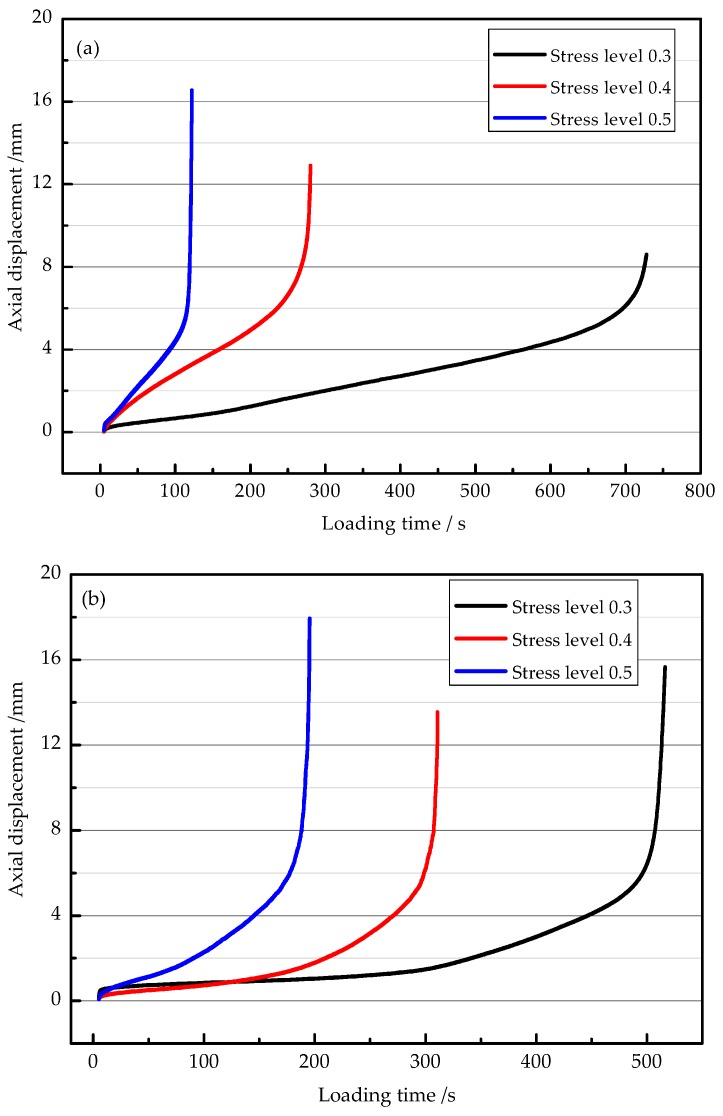

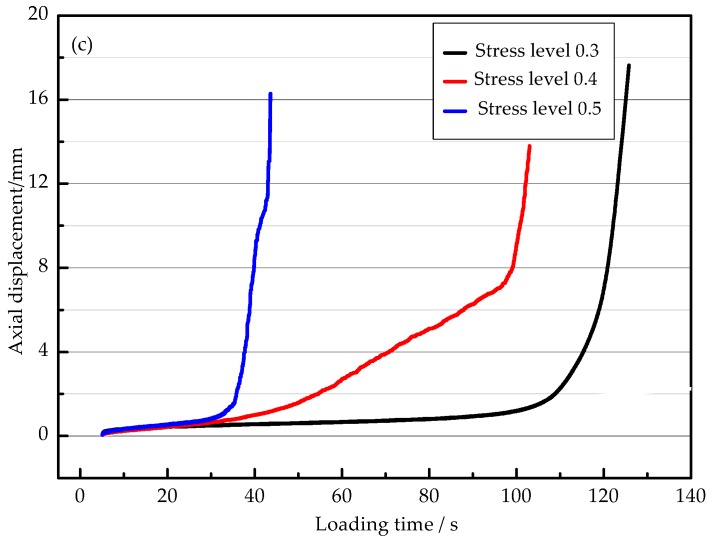
Time–displacement curves of different thickness structure combinations: (**a**) C1; (**b**) C2; and (**c**) C3.

Figure 7 shows the relationship between the axial displacement and the loading time during the loading process, at the temperature of 15 °C. The loading time is a total loading time induced by the dynamic load, which is the total time from the start to the end of loading process. The processes of the fatigue fracture of the combinations can be divided into three stages based on loading time, which are the initial stage of cracking, cracking expansion stage, and the final stage of the complete fracture. As shown in Figure 7, the initial stage of cracking for C3 under all of the stress levels is considered to be shorter in contrast to that for the other two combinations. All of the specimens deform quickly and the deformation is not significant enough to be observed in other two stages. Meanwhile, the deformations of all of the specimens present a slow and steady increase in the cracking expansion stage. In the final stage of complete fracture, deformation increases rapidly and the specimens endure complete failure. Furthermore, all of the time–displacement curves are approximately straight in the final stage. In each combination, the loading time for fatigue failure increases as the stress level decreases.

**Figure 8:**
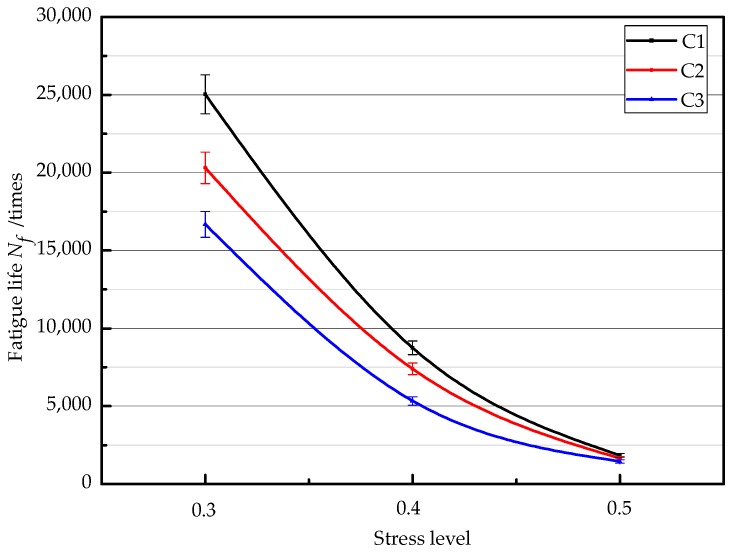
Fatigue life of different thickness structure combinations at 15 °C.

Figure 8 shows the effect of the stress levels on the fatigue life of each thickness structure combination at the temperature of 15 °C. The fatigue life of each combination increases with the decrease of the stress level. The reason is that the stiffness of the combination decreases with the increase of the stress levels. Thus, the fatigue life decreases with the increase of stress levels. Another reason is that the fatigue life is a gradual accumulation process. In the repeated cyclic changes of the long-term maximum axial load and the minimum axial load, the repeated loads exceed the ultimate stresses and strains that the specimen can bear; thus, permanent fracture damages emerge in the specimens. Therefore, the fatigue life of the combinations can decrease with the increase of the stress level.

### 3.4. Establishment of Fatigue Equation

The logarithmic fatigue life of the asphalt mixture at different stress ratios obeys the normal distribution law [25]. The relationship between stress ratio level and fatigue life is shown in Equation (4).

(4) $$\mathrm{lg}N_{f}=m-ns_{i}$$

where $N_{f}$ is the fatigue life of the asphalt mixture; $s_{i}$ is the stress ratio level; and m and n are the linear regression constant.

**Figure 9:**
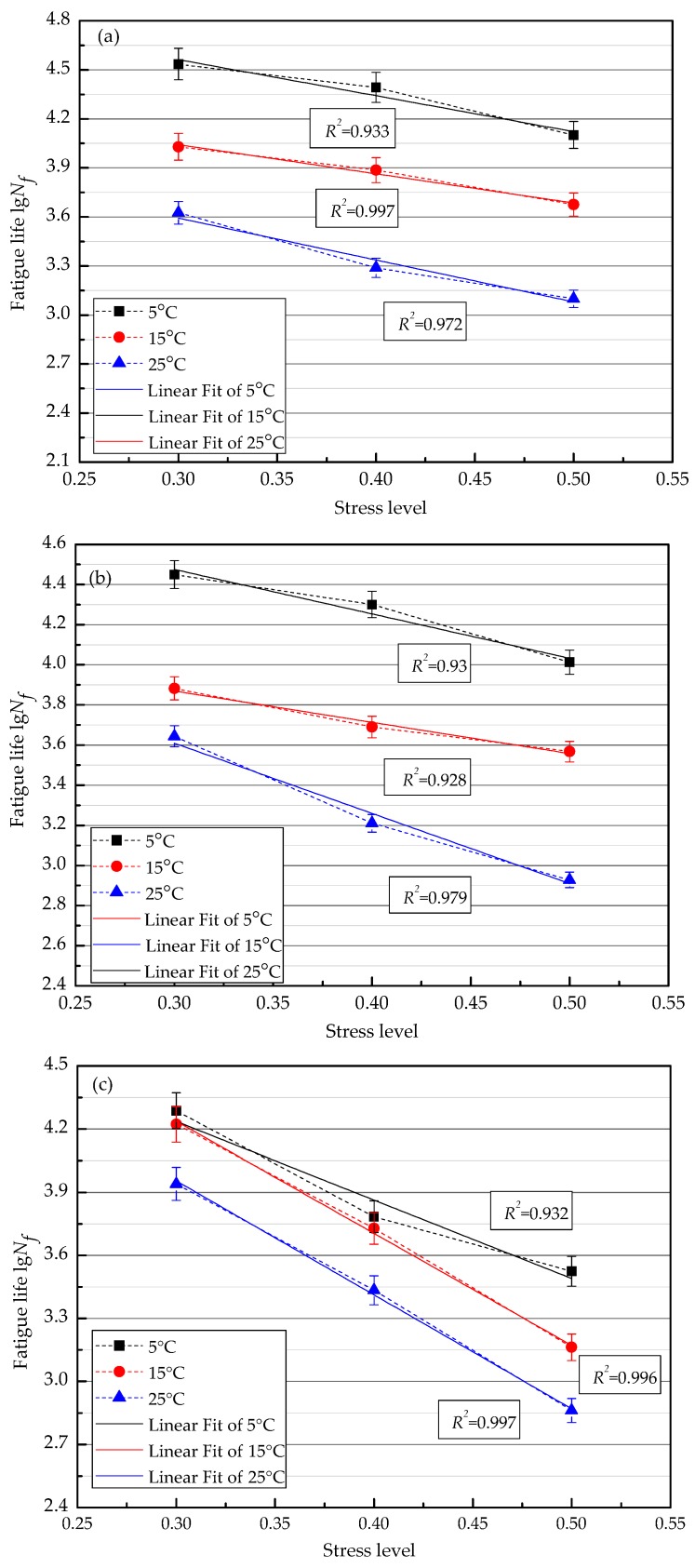
Single logarithmic fatigue curve equation for different thicknesses structure combinations under different stress ratio level: (**a**) C1; (**b**) C2; and (**c**) C3.

According to Equation (4), the single log-linear regression for the fatigue life of the combinations under three stress ratio levels are shown in Figure 9. The regression equations concerning the stress level and fatigue life are shown in Table 9. As shown in Figure 9 and Table 9, the fatigue life of the combinations decreases with the increase of the stress level. In addition, the single log-linear regression lines of the fatigue life of the combinations show the good fittings. At the same stress level, the fatigue life of the combinations increases with the decrease of the temperature. In contrast to the slope and the intercept of the regression line, the fatigue life value of the combinations is the highest and the combinations possess the best fatigue performance at the temperature of 5 °C.

### 3.5. Effects of Void Ratios on Fatigue Performance

**Figure 10:**
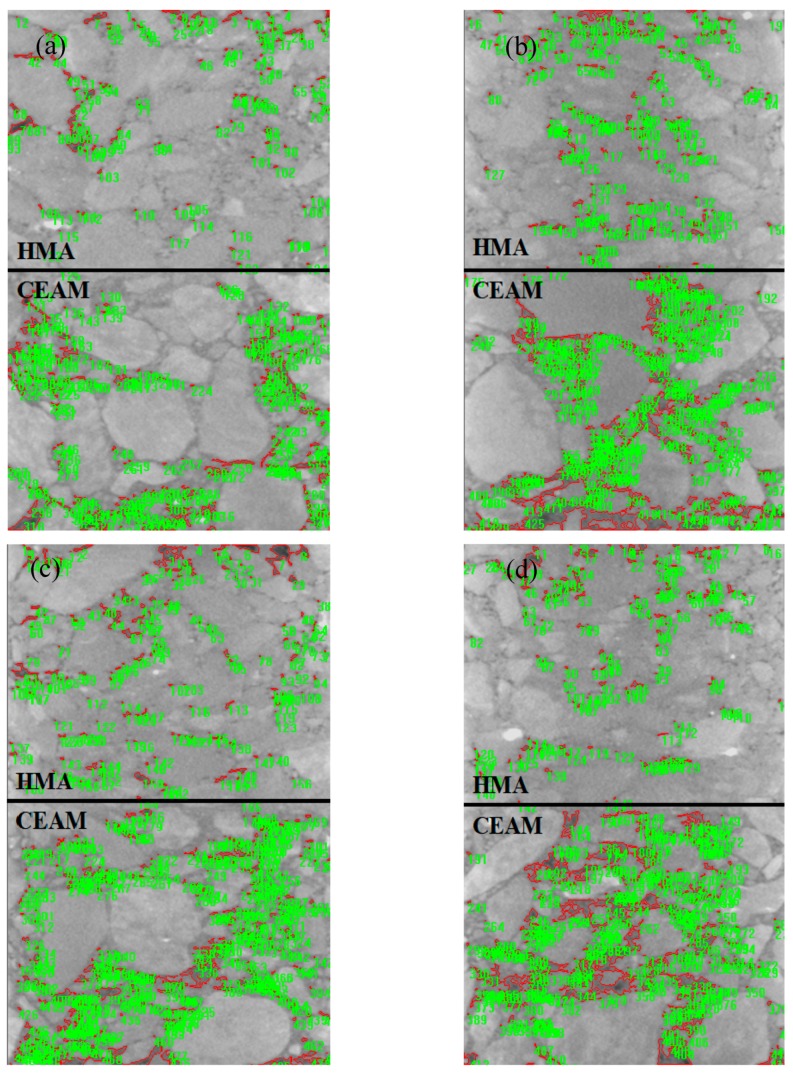
Voids recognition in CT image of C1: (**a**) void recognition in the specimen of 50 mm; (**b**) void recognition in the specimen of 100 mm; (**c**) void recognition in the specimen of 150 mm; and (**d**) void recognition in the specimen of 200 mm.

In order to study the effect of the internal voids on the fatigue performance of the combinations, an X-ray CT scanning machine was used to analyze the internal features of the C1 combination [26], whose fatigue life value is the highest. X-ray CT models were reconstructed in this work. In addition, the voids were marked and distinguished from other objects (aggregate, asphalt mortar) based on the grayscale (0–121). Therefore, software was employed to determine the grayscale threshold of the voids in the combinations. Then, the air voids can be determined by its pixel area. The typical results of the void recognition for C1 are shown in Figure 10, and the results of air voids processed by commercial software are shown in Table 10. It is clear that the voids are mainly distributed at the bottom of the specimens. In addition, the void ratios of HMA and CEAM increase first, and then decrease from 50 mm to 200 mm. The air voids of both the top layer and the bottom layer of the X-ray CT images were calculated and the average was adopted. Previous studies show that the fatigue life of the asphalt mixture increases significantly with the decrease of the air voids of the mixture [27,28]. It is because the greater air voids lead to more micro cracks in the asphalt mixture, which results in the expansion and destruction of the micro cracks under the repeated loads, which then leads to the reduction of the fatigue performance. Therefore, the double layers with thicker HMA and thinner CEAM possess a better fatigue performance. The thickness of HMA decreases with the increase of the CEAM thickness, which results in the decrease of the fatigue life of the combinations. This conclusion can be proven by the fatigue life of the C3 combination, which possesses the lowest fatigue life value.

### 3.6. Fatigue Life Prediction of Different Combinations Based on ANN

**Figure 11:**
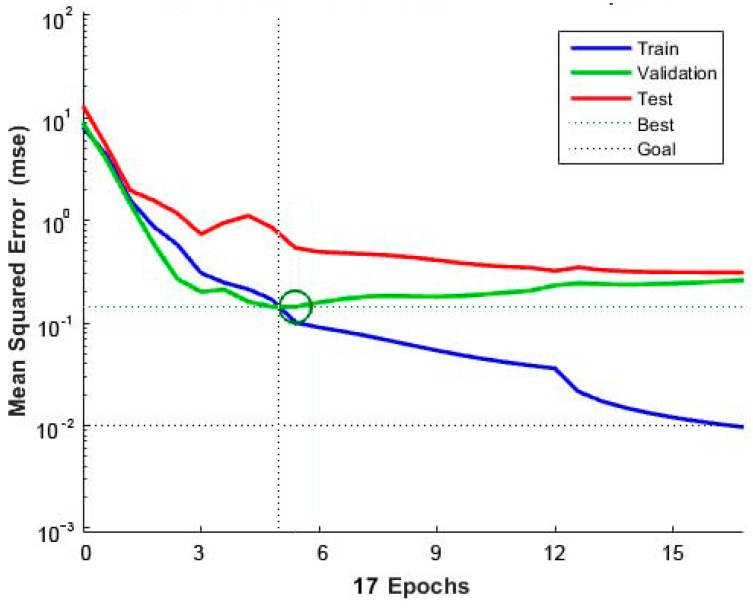
Training results of the artificial neural network (ANN).

**Figure 12:**
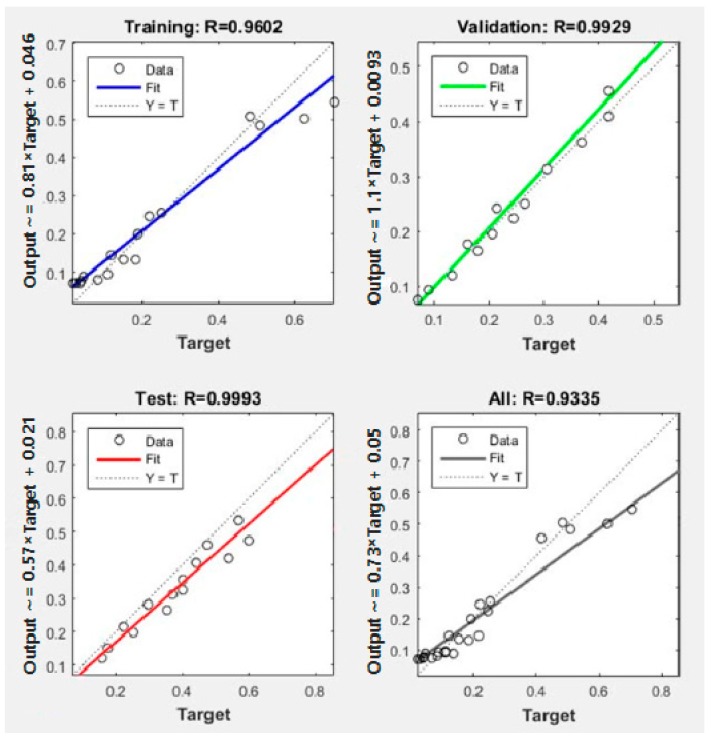
Correlations *R* between outputs and targets.

The training performance is the first step for the fatigue life prediction of different combinations, based on ANN. The training results of the ANN are shown in Figure 11. As shown in Figure 11, the mean squared error between the outputs and targets is less than 10^−1^, which means that the ANN shows good performance in the training after five epochs. Additionally, as shown in Figure 12, the correlations *R* between the outputs and the targets of training and validation are both above 0.900, which means that the errors of the outputs and targets are acceptable. Therefore, the model can be used to predict the fatigue life of other conditions, which are not tested by the three-point bending load fatigue test.

The testing performance is the second step for the fatigue life prediction of different combinations, based on ANN. The fatigue life of the combinations was predicted by the ANN in the stress levels of 0.20, 0.25, 0.30, 0.35, 0.40, 0.45, and 0.50. The results are shown in Table 11. As shown in Table 11, the fatigue life of the combinations decreases with the increase of temperature. The fatigue life of the combinations decreases with the increase of the stress level. The C1 combination (40 mm + 40 mm) possesses the highest ability to anti fatigue destroy, which is the same as the experimental results. In contrast to the test results, compared to the prediction results in Table 11, the output precision of the ANN is also acceptable. Therefore, the structure of the ANN is reasonable. With the acceptable precision, the prediction of the ANN can be used for analyzing the fatigue life of the HMA and CEAM structure combinations in asphalt pavement engineering.

## 4. Conclusions and Recommendation

This work presents the study of the fatigue performance of a double-layer asphalt pavement surface with three thickness combinations. The effect of the stress level and temperature on the fatigue performance was studied to determine the optimal thickness structure combination for the double-layer asphalt pavement surface consisting of HMA and CEAM. Additionally, the effect of the internal voids on the fatigue performance was analyzed by X-ray CT scanning. Finally, the ANN model was established to predict the fatigue life of the combinations. The following conclusions can be drawn:

(1) With the decrease of the CEAM layer thickness, the flexural tensile strength and the maximum load of the combinations increase linearly. The fatigue life of the different thickness combinations decreases with the increase of the stress level and increases with the decrease of the test temperature.
(2) The effect comparison between the stress level and the temperature shows that the 40 mm (HMA) + 40 mm (CEAM) thickness structure combination possesses the best fatigue performance. Under the controlled stress mode, the stiffness of CEAM decreases with the decrease of the temperature; and the fatigue life can be increased with the decrease of the CEAM thickness.
(3) The results of the X-ray CT test show that the air void of CEAM is greater than that of HMA; and the air voids are mainly distributed at the CEAM layer of the specimens. The air voids of the combinations increase with the increase of the CEAM thickness, which can result in the decrease of fatigue life of the combinations. Therefore, the CEAM layer thickness should be rationally selected in the design of asphalt pavement structure.
(4) The ANN was established to predict the fatigue life of the combinations. It is approximately equal to the experimental results. Therefore, the outputs of the ANN can be used to predict the fatigue life of different structures under different service conditions, in consideration of the different thickness combinations. Of course, the properties of the asphalt mixture can also influence the fatigue results, and they should be considered in the input layer of the neural network. Therefore, the properties of the mixture itself as the parameters of the ANN are recommended to consider in future research.
(5) Although CEAM is widely used in asphalt pavement for its environmental virtues, it can influence the fatigue performance of asphalt pavements as to its void characteristics. It is recommended to use HMA and CEAM combinations and to consider their rational thickness structure combination in CEAM asphalt pavement design.

## Figures and Tables

**Table 1:** Properties of asphalt binder.

Properties	Unit	Specification [20]	Test Results
Penetration (25 °C, 5 s, 100 g)	0.1 mm	60–80	72
Softening point (Ring-and-ball method)	°C	≥46	52.3
Ductility at 15 °C	cm	≥40	51
Solubility	%	≥99.5	99.71
Density at 15 °C	g/cm^3^	Measured	1.036
Wax content (distillation method)	%	≤2.2	1.7
Flash Point (COC)	°C	≥260	305

**Table 2:** Properties of ordinary Portland cement.

Fineness/(%, 80 μm)	Water Consumption of Normal Consistency/%	Stability (Boiled Method)	Setting Time/min		Compressive Strength/MPa		Flexural Strength/MPa	
			Initial	Final	3 d	28 d	3 d	28 d
2.4	23.2	qualified	143	201	28.7	52.5	6.2	9.3

**Table 3:** Properties of emulsified asphalt.

Properties		Unit	Specification [20]	Test Results
Particle charge type		-	Cationic	Cationic
The remaining percentage on 1.18 mm sieve		%	≤0.1	0.03
Wrap ratio with coarse aggregate		-	≥2/3	>2/3
Evaporation residue	Residue content	%	63	≥55
	Penetration (5 s, 100 g, 25 °C)	0.1 mm	75	50–300
	Ductility (15 °C)	cm	47	≥40
Storage stability at room temperature	1 d	%	0.63	≤1
	7 d	%	3.24	≤5

**Table 4:** Properties of aggregate.

Properties	Unit	Specification [21]	Test Results
Apparent density	g/cm^3^	≥2.50	2.805
Crushing value	%	≤20	13.2
Wear loss in Los Angeles	%	≤24	18.3

**Table 5:** Properties of mineral fillers.

Properties		Unit	Specification [21]	Test Results
Apparent density		g/cm^3^	≥2.50	2.826
Moisture content		%	≤1	0.45
Particle size	<0.6 mm	%	100	100
	<0.15 mm		90–100	98.6
	<0.075 mm		75–100	83.7
Appearance		-	No lumps	No lumps
Hydrophilic coefficient		-	<1	0.6

**Table 6:** Properties of hot mix asphalt (HMA).

Properties	Unit	Test Results
Optimum asphalt content	%	4.6
Marshall stability	kN	12.2
Flow value	mm	3.28
Dynamic stability	times/mm	942
Residual stability	%	87

**Table 7:** Properties of cement emulsified asphalt mixture (CEAM).

Properties	Unit	Test Results
Emulsified bitumen content	%	8.0
Cement content	%	2.0
Marshall stability	kN	10.3
Flow value	mm	3.23
Dynamic stability	times/mm	1088
Residual stability	%	83

**Table 8:** The parameters of industrial X-ray computed tomography (X-ray CT).

Parameters	Results
Maximum tube voltage	225 kV
Maximum tube current	3.0 mA (D)/1.0 mA (T); 320 W (D)/64 W (T)
Operation mode	Cone beam scanning and digital imaging
Magnification	200 times (D)/100 times (T)
Specimen size	Length: 50 mm–100 mm; Width: 50 mm; Height: 80 mm
Pixel size	1024 × 1024 (200 × 200 µm^2^)
Filter combination	Al: 1 mm; Cu: 1 mm; Fe: 0.5 mm; Sn: 0.5 mm
Dimension precision	<5 µm

**Table 9:** Stress ratio level—fatigue life regression equation of each combination.

Combinations	Temperature (°C)	Regression	$R^{2}$
C1 (40 mm + 40 mm)	5	$\mathrm{lg}N_{f}=-2.0s_{i}+5.153$	0.934
	15	$\mathrm{lg}N_{f}=-1.6s_{i}+4.480$	0.997
	25	$\mathrm{lg}N_{f}=-3.15s_{i}+4.573$	0.972
C2 (30 mm + 50 mm)	5	$\mathrm{lg}N_{f}=-2.25s_{i}+5.153$	0.932
	15	$\mathrm{lg}N_{f}=-1.65s_{i}+4.357$	0.928
	25	$\mathrm{lg}N_{f}=-3.65s_{i}+4.723$	0.979
C3 (20 mm + 60 mm)	5	$\mathrm{lg}N_{f}=-3.85s_{i}+5.403$	0.95
	15	$\mathrm{lg}N_{f}=-5.3s_{i}+5.823$	0.992
	25	$\mathrm{lg}N_{f}=-5.4s_{i}+5.570$	0.997

**Table 10:** Air voids of the mixtures at different depths.

Different Depths of the Specimens/mm	Air Voids of the Mixtures/%	
	HMA	CEAM
50	1.9	7.7
100	2.4	8.5
150	2.6	8.6
200	2.2	8.3

**Table 11:** Normalized experimental results and prediction results of the combinations.

Temperature (×40)/°C	Stress Level	Fatigue Life of C1 (×40,000) *N_f_*/Times		Fatigue Life of C2 (×40,000) *N_f_*/Times			Fatigue Life of C3 (×40,000) *N_f_*/Times		
		Test Data	Prediction		Test Data	Prediction		Test Data	Prediction
0.125	0.20		0.878			0.766			0.560
	0.25		0.862			0.762			0.551
	0.30	0.857	0.849		0.704	0.702		0.484	0.479
	0.35		0.450			0.444			0.383
	0.40	0.219	0.226		0.191	0.196		0.152	0.148
	0.45		0.120			0.106			0.097
	0.50	0.124	0.093		0.111	0.092		0.084	0.091
0.375	0.20		0.705			0.628			0.553
	0.25		0.664			0.516			0.478
	0.30	0.626	0.615		0.508	0.513		0.417	0.416
	0.35		0.441			0.304			0.290
	0.40	0.245	0.234		0.185	0.179		0.134	0.142
	0.45		0.111			0.087			0.074
	0.50	0.046	0.043		0.041	0.043		0.036	0.040
0.625	0.20		0.500			0.458			0.350
	0.25		0.493			0.386			0.283
	0.30	0.337	0.343		0.251	0.254		0.218	0.236
	0.35		0.378			0.145			0.117
	0.40	0.118	0.144		0.089	0.093		0.068	0.074
	0.45		0.082			0.071			0.061
	0.50	0.026	0.024		0.021	0.018		0.018	0.010
